# Impact of subclinically hypocalcemic stress on the plasma metabolomic profile of dairy goats

**DOI:** 10.5713/ab.24.0567

**Published:** 2025-01-24

**Authors:** Jia Zhang, Dan Yang, Yafei Zeng, Kaini Guo, Jiajian Zhang, Yan Huang, Yuzhen Sui, Qimin Liu, Xiaoxuan Mo, Chenxu Zhao, Jianguo Wang

**Affiliations:** 1 College of Veterinary Medicine, Northwest A&F University, Yangling, China

**Keywords:** Guanzhong Dairy Goat, Lipidomics, Subclinically Hypocalcemic Stress, Transition Period, Untargeted Metabolomics

## Abstract

**Objective:**

This study aimed to explore novel aspects of disease prevention and control in Guanzhong dairy goats through the application of metabolomics and lipidomics.

**Methods:**

In this study, plasma samples were collected from 96 primiparous Guanzhong dairy goats with similar body condition scores (2.75±0.15, mean±standard deviation) on the day of calving. The aim was to identify key differences in metabolite expression between diseased and healthy animals using metabolomics and lipidomics.

**Results:**

Twenty-three differential metabolites and 30 differentially altered lipids were identified, which were associated with various metabolic pathways, including phenylalanine and tyrosine metabolism, aminoacyl-tRNA biosynthesis, and glycerophospholipid metabolism. Our research revealed significant differences in the regulation of calcium-related hormones and associated metabolites between subclinical hypocalcemic and healthy dairy goats. Specifically, parathyroid hormone and aspartate aminotransferase were positively correlated in the healthy group and negatively correlated in the subclinical hypocalcemic group.

**Conclusion:**

The identification of phenylalanine and phosphatidylserine as potential biomarkers for subclinical hypocalcemia in dairy goats offers a novel approach to managing this condition, potentially transforming prevention and control strategies in the dairy goat industry.

## INTRODUCTION

During the peripartum period, dairy animals frequently encounter a negative energy balance) due to insufficient energy intake relative to expenditure [1,2]. This metabolic challenge notably increases the susceptibility to nutritional metabolic disorders, such as subclinical hypocalcemia (SCHC) [3,4], which can subtly undermine calcium homeostasis and impact health by reducing milk yield and reproductive efficiency [5,6]. SCHC is particularly insidious, as its effects frequently evade clinical detection despite notable deviations in serum calcium concentrations [7], complicating diagnosis and monitoring in practical settings. Therefore, establishing clear diagnostic criteria and effective early intervention strategies for SCHC is crucial not only for enhancing the welfare of dairy animals but also for ensuring sustained production efficiency.

While much research on dairy goats has focused on proteomics to improve the specificity of subclinical mastitis detection and aid in management decisions during the peripartum period [8], there remains a significant gap in knowledge regarding the transition phase of dairy goats. Metabolomics, a relatively new yet impactful technology in this field, has made substantial contributions across various scientific disciplines. Its ability to analyze the metabolic profiles of organisms has been instrumental in advancing our understanding of human tumors [9], plant science [10], and immunological responses [11]. Targeted metabolomics, particularly lipidomics, offers a high level of precision in examining metabolite clusters associated with specific metabolic pathways [12]. Integrating metabolomics into goat research presents an opportunity to gain new insights into the health management of dairy goats, especially during critical transition periods. Metabolomics holds promise in addressing the diagnostic challenges of SCHC, which often presents without clinical signs, by potentially discovering biomarkers and revealing pathomechanisms that are not apparent through clinical examination [13]. The proven utility of metabolomics across diverse fields underscores its potential to enhance our understanding and improve the precision of veterinary and medical practices.

In this study, we utilized insights from metabolomics and lipidomics to delve into the pathogenesis of hypocalcemia in dairy goats, an area that, despite its significance, has received less scrutiny compared to dairy cows. We aimed to identify the metabolomic signatures associated with SCHC in goats, hypothesizing that specific metabolite perturbations could indicate the presence of this condition. By analyzing plasma samples from both clinically healthy goats and those diagnosed with SCHC, we employed a dual approach of untargeted metabolomics to survey a broad metabolic landscape and targeted lipidomics to quantify known metabolites of interest. Our findings not only enhance the current understanding of the metabolic underpinnings of SCHC but also provide novel biomarkers for early detection, prevention, and treatment strategies, ultimately contributing to enhancing health and productivity in dairy goat populations.

## MATERIALS AND METHODS

### Animals, location, and study design

All animal experiments were approved by the Ethics Committee on the Use and Care of Animals at Northwest A&F University (Ethical Approval number: 2021046). The experiment was conducted from January to March 2019 at the experimental site, at a temperature of 11±2°C and an average relative humidity of 57±4%. The subjects were China Guanzhong dairy goats, which are widely distributed in Shaanxi Province. All trials were conducted at Northwest A&F University experimental farm in Western China (106°55′ 57″ E,34°48′ 41″ N). Ninety-six primiparous Guanzhong dairy goats were homogeneous in age (1 to 2 years), body weight (61±4.8 kg), and body condition score (2.75±0.15), and were used as the initial experimental animals. In September, the farm goats involved in this study were given estrous synchronized such that kidding occurred in February. All goats were fed the same diet offered twice daily at 07:30 and 15:30 h ad libitum as a total mixed ration, with a mean dry matter intake of 1.48±0.06 kg/d. The diets met the nutrient requirements according to the Nutrient Requirements of Small Ruminants [14].

### Blood sample collection and biochemical analyses

The blood samples (10 mL) were collected from individual goats at −3, −2, −1, 0 (partum), +1, +2, and +3 weeks from delivery from the jugular vein using vacutainer tubes containing sodium heparin (Becton-Dickinson, Franklin Lakes, NJ, USA). At antepartum, the blood samples for each goat were collected before feeding in the morning. At postpartum, the blood samples were collected after the milking operations, but before feeding in the morning. All the tubes for plasma collection were immediately placed in an ice bath. The blood samples were collected (10 mL) aseptically into Heparin Sodium collection tubes, then centrifuged at 2,000×g for 10 min at 4°C, and the supernatant plasma was packaged before storage at −80°C until further use. Plasma calcium (kit no. CA590; Arsenazo III method) concentrations were determined using a Hitachi auto-analyzer (Hitachi, Fukuoka, Japan). A total of 96 primiparity Guanzhong dairy goats were tested in this experiment of which 18 were selected, among which 9 goats with no clinical symptoms of hypocalcemia with calcium concentrations between 1.5 and 2.2 mmol/L were regarded as SCHC group, and goats with no clinical signs and calcium concentration > 2.2 mmol/L were regarded as healthy (HEAL) group.

The plasma β-hydroxybutyrate ([BHBA], no. RB1007, enzymatic method; Randox Laboratories, Crumlin, UK), the non-esterified fatty acids ([NEFA], no. FA115, colorimetric method; Randox Laboratories), and calcium (Ca, no. CA590, Arsenazo III method) concentrations were analyzed by using commercial kits (Randox Laboratories) and a Hitachi auto-analyzer (Hitachi, Fukuoka, Japan). The levels of glutamic oxaloacetic transaminase (AST), alkaline phosphatase, lactate dehydrogenase (LDH), NEFA, total cholesterol (TC), globulin (GLB), low-density cholesterol (LDL), creatinine (CREA), high-density cholesterol, glucose, triglyceride, UREA, albumin, aminotransferase, γ-glutamyl transpeptidase, cholinesterase, total protein in plasma were determined with a chemical autoanalyzer (Hitachi 7060, Hitachi). The concentrations of calcitonin (CT) (MM-750610), parathormone (MM-750620), and 1,25-dihydroxy vitamin D3 (MM-750590), in plasma samples, were measured by double antibody sandwich method with goat-specific ELISA kits (Meimian Biotechnology, Yancheng, China) and the Bio-Rad 680 microplate reader (Bio-Rad, Hercules, CA, USA). Optical density (OD) values were recorded at 450 nm, and results were calculated by comparing sample OD to a standard curve.

### Metabolome extraction

Samples were prepared according to a previously described method [15]. Briefly, a 50 μL sample was mixed with 200 μL of ice-cold 80% methanol in water, and incubated for 30 min at 1,500 rpm and 4°C; then centrifuged for 10 min at 16,000×g and 4°C. The supernatant was removed into a clean 1.5 mL centrifuge tube and dried using a SpeedVac. The dried extracts were redissolved with 1% acetonitrile in water and the liquid in the upper layer was collected for the liquid chromatography triple quadrupole mass spectrometry analysis.

### Untargeted metabolomics analysis

Untargeted metabolomics were conducted by Lipid ALL Technologies Company Limited as described previously [15]. Briefly, the metabolites were separated on an Acquity UPLC HSS T3 1.8 μm, 2.1×100 mm column (Waters) using ultra performance liquid chromatography ([UPLC], Agilent 1290 II; Agilent Technologies, Santa Clara, CA, USA) and analyzed on a Quadrupole-time-of-flight mass spectrometry (MS) (5600 Triple TOF Plus; AB Sciex, Framingham, MA, USA). The MS parameters used for the detection were electrospray ionization (ESI) source voltage +5.5 kV in positive ion mode, and −4.5 kV in negative ion mode; vaporizer temperature, 500°C; drying gas (N_2_) pressure, 50 psi; the scan range was 60 to 800 m/z. The information-dependent acquisition mode was used for tandem mass spectrometry (MS/MS) analyses of the metabolites. The collision energy was set at 35±15 eV and both the data acquisition and processing were performed using the Analyst TF 1.7.1 Software (AB Sciex). Detected ions were extracted into Excel as a two-dimensional matrix containing mass-to-charge ratio (m/z), retention time, and peak areas using MarkerView 1.3 (AB Sciex). Thereafter, the isotopic peaks were filtered. The MS/MS data were extracted, and a comparison was performed with the metabolites database (AB Sciex), the Human Metabolome Database (https://hmdb.ca/), METLIN using the PeakView 2.2 (AB Sciex), and the standard references were used for annotating the ion ID.

### Targeted lipidomics analysis

Lipids were extracted from serum (20 μL) using a modified Bligh and Dyer’s extraction procedure (double rounds of extraction) and dried in the SpeedVac under OH mode [15]. Before carrying out the analysis, the lipid extracts were resuspended in chloroform: methanol 1:1 (vol/vol) spiked with appropriate internal standards. The lipidomic analyses were carried out on an Exion UPLC system coupled with a QTRAP 6500 PLUS system (Sciex, Framingham, MA, USA) under an ESI mode as described previously unless otherwise stated. All the quantification experiments were conducted using an internal standard calibration. The various lipid types measured included acylcarnitine, cholesteryl esters (CE), ceramides, diacylglycerols (DAG), free fatty acids, monosialogangliosides (GM3), phosphatidic acids (PA), phosphatidylcholines (PC), lyso-PC (LPC), phosphatidylethanolamines (PE), phosphatidylglycerols (PG), phosphatidylinositols (PI), phosphatidylserines (PS), sphingosine-1-phosphate (S1P), sphingomyelins (SM), and triacylglycerols (TAG).

In brief, the polar lipids were separated on a Phenomenex Luna Silica 3-μm column (i.d. 150×2.0 mm) using the mobile phase A (chloroform:methanol:ammonium hydroxide, 89.5:10:0.5) and mobile phase B (chloroform:methanol: ammonium hydroxide:water, 55:39:0.5:5.5) at a flow rate of 270 μL/min and column oven temperature at 25°C. The individual polar lipid species were then quantified by reference to the spiked internal standards, which included PC-14:0/14:0, PE14:0/14:0, d31-PS-16:0/18:1, PS-17:0/20:4, PA-17:0/17:0, PG-14:0/14:0, GluCer-d18:1/8:0, Cer-d18:1/17:0, C14:0-BMP, S1P-d17:1, Sph-d17:1, SM-d18:1/12:0, LPC-17:0, LPE-17:1, LPI-17:1, LPA-17:0, LPS-17:1 obtained from Avanti Polar Lipids (Alabaster, AL, USA) and PI-8:0/8:0 from Echelon Biosciences Inc (Salt Lake City, UT, USA). The GM3 species were quantified using GM3d18:1/18:0-d3 from Matreya LLC (State College, PA, USA).

The glycerol lipids including DAG and TAG were quantified using a modified version of reverse-phase high-performance liquid chromatography/multiple reaction monitoring [15]. The neutral lipids were separated on a Phenomenex Kinetex-C18 2.6 μm column (i.d. 4.6×100 mm) using an isocratic mobile phase containing chloroform:methanol:0.1 M ammonium acetate 100:100:4 (vol/vol/vol). The levels of different short-, medium-, and long-chain TAG were calculated about the spiked internal standards of TAG (14:0)3-d5, TAG (16:0)3-d5, and TAG (18:0)3-d5 obtained from the CDN isotopes, respectively. The DAG was quantified using the d5-DAG16:0/16:0 and d5-DAG18:1/18:1 as internal standards (Avanti Polar Lipids). The free cholesterol and CE were quantitated with d6-cholesterol and d6-C18:0 cholesteryl ester (CDN isotopes) as the internal standards under atmospheric pressure chemical ionization. The plasma lipid levels were expressed in nanomoles per liter.

### Data processing and statistical analyses

The biochemical data were statistically analyzed using GraphPad Prism 8.0 (GraphPad Software Inc., Boston, MA, USA). The data were analyzed with unpaired t-tests and results were expressed as means±standard deviation.

Untargeted metabolomics and targeted lipidomics data were log2-transformed and Pareto-scaled before analysis. Multivariate analysis was performed using MetaboAnalyst 4.0 (http://www.metaboanalyst.ca) comprising the supervised orthogonal partial least squares discriminant analysis (OPLS-DA) and the pathway enrichment analyses. The intergroup differences were further determined using OPLS-DA. The OPLS-DA models were validated based on the interpretation of variation in Y (R^2^Y) and the prediction ability based on the model (Q_2_) in cross-validation and permutation tests by applying 200 iterations. Differentially altered metabolites were screened using p-values (p<0.05) from the unpaired t-test. Heatmap visualizations were performed using the R package “heatmap” and the clustering method used “complete” (version 4.1.3, http://www.R-project.org). The pathway enrichment analyses were performed using MetaboAnalyst 4.0 (http://www.metaboanalyst.ca) based on the differentially altered metabolites. Spearman correlations between untargeted metabolomics and targeted lipidomics data were analyzed in R using the “Corr. test.”

## RESULTS

### Plasma biochemical data

The biochemical data are summarized in Table 1. Significant variations in plasma biochemical indices, including BHBA, AST, and Ca, were observed during the peripartal period. Notably, calcium levels in the SCHC group were significantly lower than those in the HEAL group (p<0.05). Additionally, the SCHC group exhibited significantly higher plasma BHBA and AST, which are primarily associated with liver function and commonly used to evaluate liver disease (p<0.01), along with increased levels of LDH, NEFA, TC, GLB, and LDL (p<0.5).

**Figure 1:**
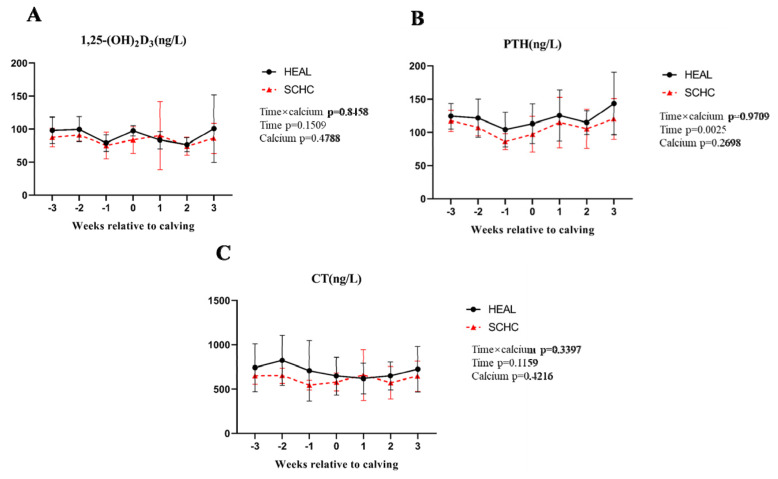
Changes in calcium-regulating hormones were analyzed in subclinical low-calcium dairy goats from three weeks before parturition to three weeks after parturition. The solid black line represents healthy dairy goats, and the dotted red line represents subclinical low-calcium dairy goats. (A) 1,25-dihydroxyvitamin D3 (1,25(OH)_2_D_3_) (ng/L), (B) parathyroid hormone (PTH) (ng/L), (C) calcitonin (CT) (ng/L). The changes of the above three indexes from 3 weeks before to 3 weeks after delivery and on the day of delivery of dairy goats in two groups were monitored (n = 9). HEAL, healthy; SCHC, subclinical hypocalcemia.

The study analyzed changes in calcium-regulating hormones, including calmodulin, from three weeks before parturition to three weeks postpartum. Levels of CT and 1,25 (OH)_2_D_3_ peaked at parturition (as shown in Figures 1A, 1C), with subsequent recovery observed in the week following. Moreover, a decrease in plasma parathyroid hormone (PTH) levels was noted from −3 weeks to +3 weeks postpartum (Figure 1B).

### Multivariate statistical analysis of metabolomics

**Figure 2:**
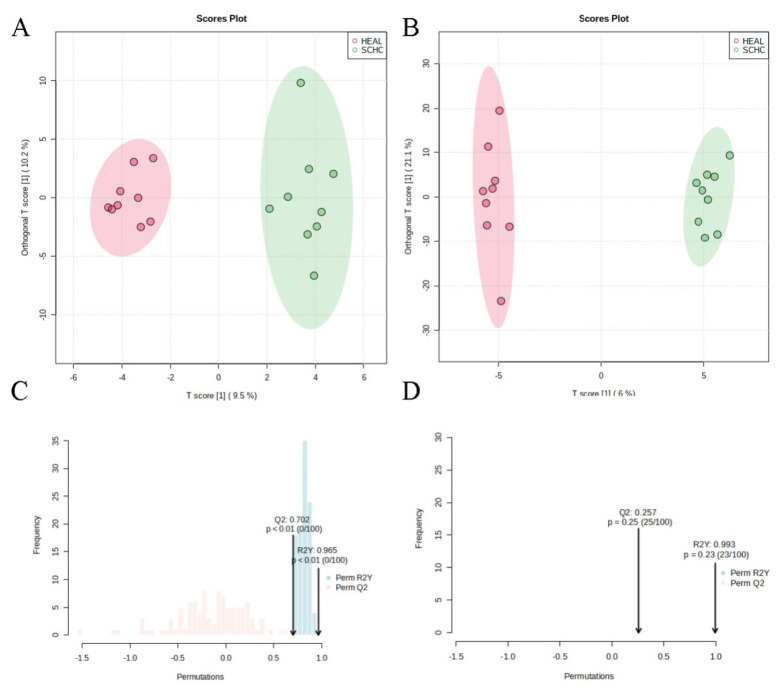
Pattern analysis of data from the metabolic profiles of plasma and lipidomics by UPLC-Q-TOF/MS. (A) OPLS-DA model (R^2^Y=0.965 and Q^2^=0.702), (B) OPLS-DA model (R^2^Y = 0.993 and Q2 = 0.257), (C) and (D) supervised model. The metabolomics data revealed the classification of each sample within the group, which was distinguished between the HEAL group and the SCHC group according to the OPLS-DA model. HEAL, healthy; SCHC, subclinical hypocalcemia; UPLC, ultra performance liquid chromatography; Q-TOF/MS, Quadrupole-time-of-flight/mass spectrometry; OPLS-DA, orthogonal partial least squares discriminant analysis.

Untargeted metabolomics was used to identify and classify a total of 23 distinct metabolites. According to the OPLS-DA model (Figure 2A) (R^2^Y = 0.965 and Q^2^ = 0.702), the metabolomics data revealed the classification of each sample within the group, which was distinguished between the HEAL group and the SCHC group. A total of 30 lipids were identified by targeted lipidomics. The OPLS-DA model (Figure 2B) demonstrated the distinction between the HEAL and SCHC groups (R^2^Y = 0.993 and Q^2^ = 0.257), indicating their separation. The OPLS-DA model is a supervised model (Figures 2C, 2D).

**Figure 3:**
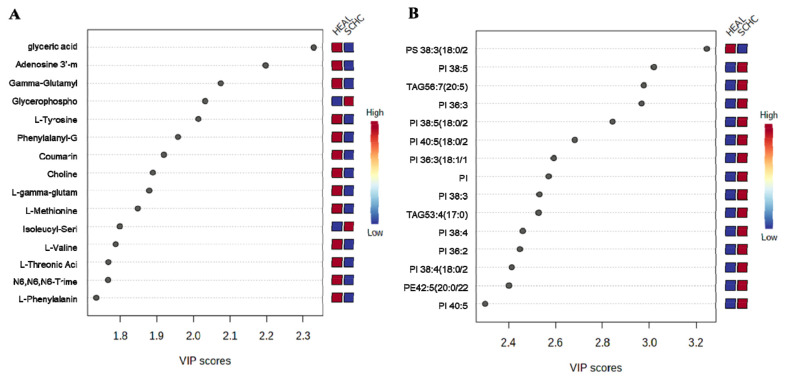
Variable importance in projection (VIP) scores for the top 15 metabolites. (A) and lipid metabolites (B) were used to differentiate the HEAL group (n = 9) and SCHC group (n = 9). VIP scores are derived from OPLS-DA analysis performed at each time point. VIP scores≥1.0 were considered significant when selecting metabolites for the final model. The column to the right of each figure displays variations in metabolite peak intensities. HEAL, healthy; SCHC, subclinical hypocalcemia; OPLS-DA, orthogonal partial least squares discriminant analysis.

The high variable importance in projection (VIP) scores indicated that the metabolites contributed significantly to the group separation. The VIP score plot highlighted the top 15 metabolites (Figure 3) based on the VIP value in the OPLS-DA model (VIP>1). Glycerophosphocholine acid, Isoleucyl-Serine, Isobutyrylglycine, and Isoleucyl-Alanine were higher in the SCHC group than in the HEAL group (Figure 3A), consistent with the lipid metabolite score plot showing that most lipid metabolites were higher in the SCHC group than in the HEAL group (Figure 3B). Among these lipid metabolites, PS were significantly lower than HEAL, and others such as PI, TAG, and PE were higher than HEAL. Among the different changed lipids, 1 LPA, 1 PC, 2 GM3, 2 PE, 3 PS, 3 TAG, and 16 PI changed greatly between the two groups, with significant increases concentrated in 4 PI and 1 PS in the SCHC group compared to the HEAL group (p<0.05).

### Screening of differential metabolites

In the SCHC group, amino acids (isoleucine-alanine, isoleucine-serine, iso butyryl glycine, valine, gamma-glutamic acid, phenylpropanylglutamic acid, phenylalanine, tyrosine, threonine, N6, N6, N6-trimethyl-l-lysine, phenylalanyl serine, sarcoline), choline (glycerophospholyl choline, choline), esters (coumarin, hydroxyphenyl fat), vitamin (riboflavin), oleic acid (glyceric acid), adenosine (adenosine triphosphate) levels were significantly lower than those in the HEAL group (p<0.05).

**Figure 4:**
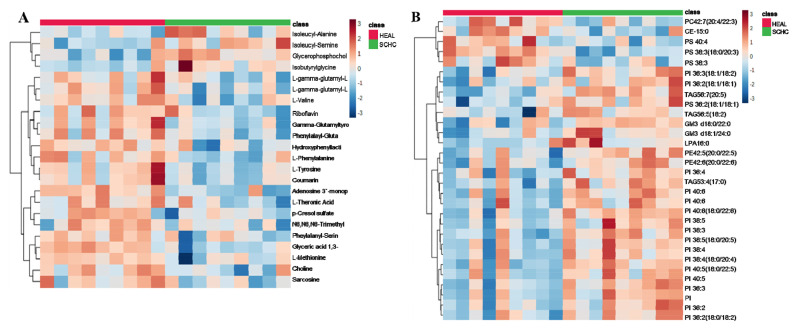
Heat map of the differential metabolites. Differentially expressed genes for (A) untargeted metabolomics, (B) lipid metabolomics.

**Figure 5:**
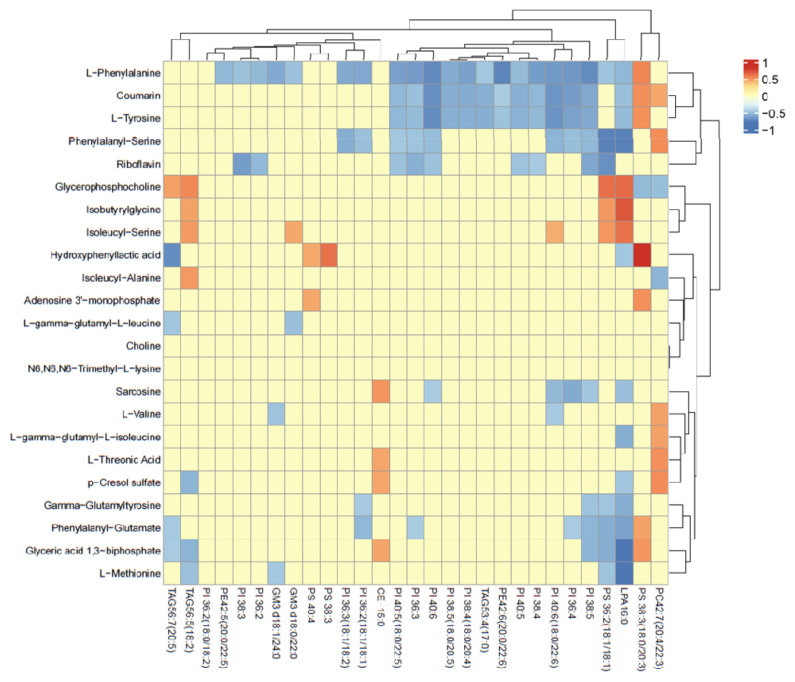
Correlation of untargeted metabolomics and lipid metabolomics in the organism of healthy dairy goats. Red dots represent metabolites that were increased in the SCHC group and blue dots represent metabolites that were decreased in the SCHC group. SCHC, subclinical hypocalcemia.

In contrast, the SCHC group had significantly higher plasma concentrations of isoleucine alanine, isoleucine serine, and glycerophosphophenol (p<0.05). Lipidomics results showed that most lipids were significantly higher in the SCHC group than in the HEAL group, especially PI (p<0.05), which was significantly lower in the HEAL group (p<0.05). Exploring the correlation between untargeted metabolites and lipid metabolites (Figure 4). The correlation heat map observed that the lipid metabolite PI, with the most substantial change, was negatively correlated with phenylalanine, tyrosine, and phenylalanine-serine, and positively correlated with glycerophosphate (p<0.05) (Figure 5).

### Differential metabolite pathways and analysis

**Figure 6:**
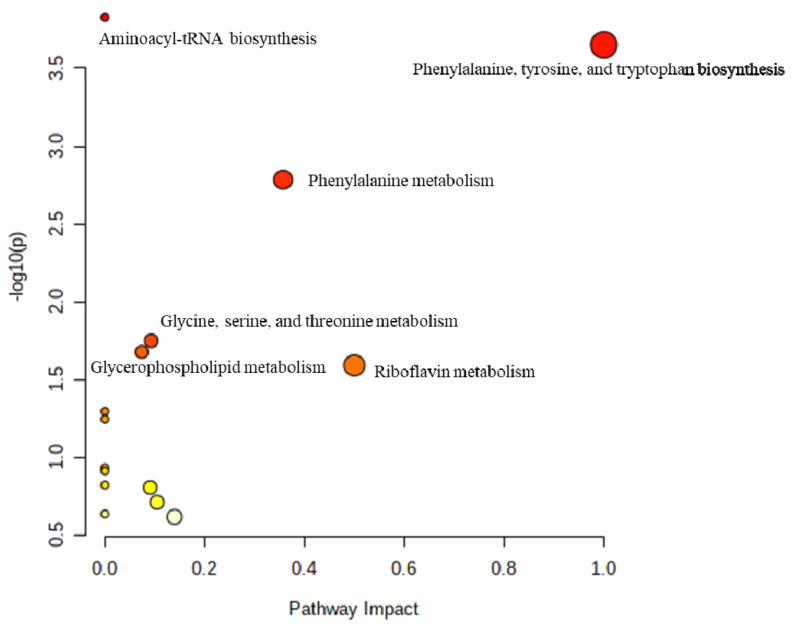
Enriched bubble diagram of differential metabolite pathways (bubble size is proportional to the degree of influence of each pathway; bubble color indicates the significant degree of influence, from the highest [red] to the lowest [white]). Metabolic pathway analysis using MetaboAnalyst 4.0. The circles represent the different metabolic pathways. The darker circles indicate significant changes for specific metabolites in the corresponding pathway, whereas the size of the circle corresponds to the pathway impact score.

**Figure 7:**
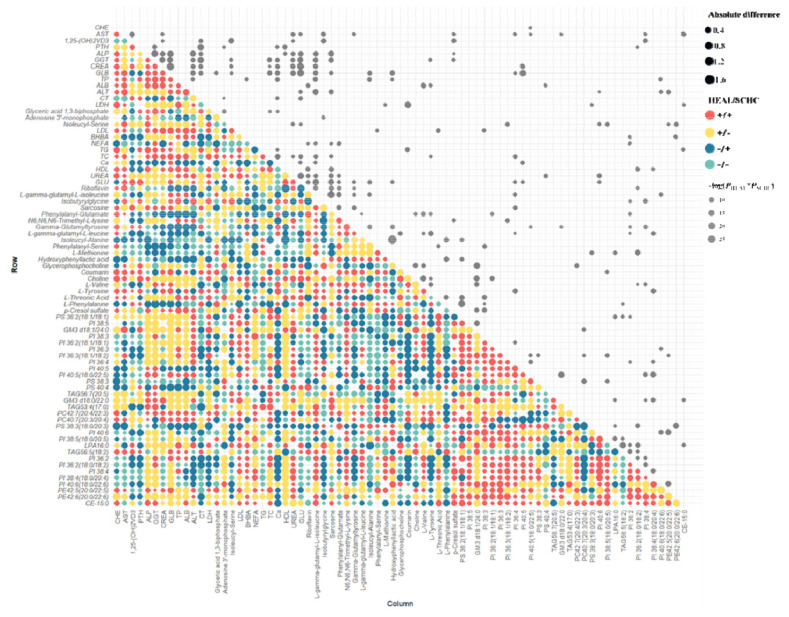
Differences in the Spearman correlations between biochemical indicators and metabolites in SCHC vs. HEAL. Biochemical indicators and metabolites relationships differ in SCHC relative to control metabolites. Features are grouped by hierarchical clustering. The size of the dots represents the absolute value of the difference, and the color represents the directions of correlations in SCHC and HEAL, respectively. 1,25(OH)_2_VD_3_, PTH, and Phe were among those differentially correlated with PI-derived metabolites in SCHC and HEAL. SCHC, subclinical hypocalcemia; HEAL, healthy; 1,25(OH)_2_D_3_, 1,25-dihydroxy vitamin D_3_; PTH, parathyroid hormone; PI, phosphatidylinositols.

The relative variation of differentially altered metabolites during the peripartal period was displayed as heat maps within the metabolic network. To further elucidate the changes in metabolic processes during this period, pathway enrichment was analyzed using significantly altered metabolites (Figure 6). Additionally, we assessed the correlation between each metabolite pair in SCHC compared to HEAL to identify co-varying biochemical indicators relevant to SCHC (Figure 7). These identified patterns of putative biochemical indicators that were differential in SCHC. For example, PTH had a positive correlation with PI-derived metabolites in SCHC.

**Figure 8:**
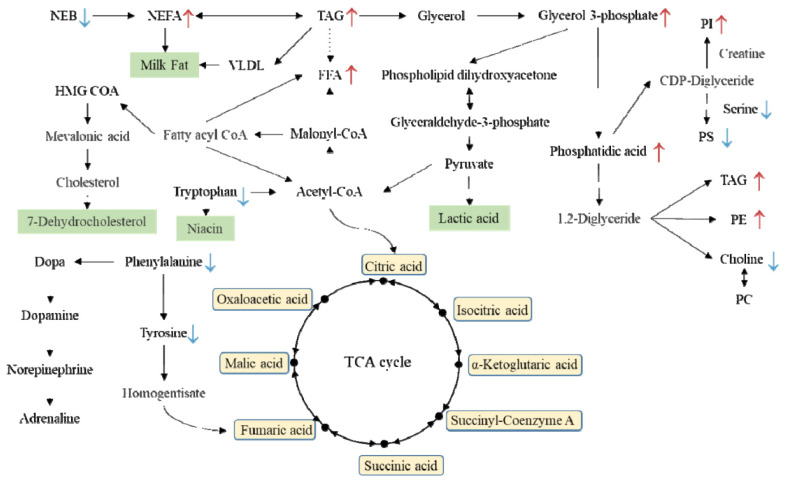
Metabolite associations between the HEAL group and SCHC group. The presence of a blue downward-pointing arrow indicates a decrease in the concentration of the substance within the SCHC group, whereas a red upward-pointing arrow signifies an increase in the substance’s concentration within the SCHC group. NEB, negative energy balance; NEFA, non–esterified fatty acids; TAG, triacylglycerols; PI, phosphatidylinositols; PS, phosphatidylethanolamines; PC, phosphatidylcholines; TCA, tricarboxylic acid; HEAL, healthy; SCHC, subclinical hypocalcemia.

Differentially altered metabolites were analyzed through the Kyoto encyclopedia of genes and genomes (KEGG) Metabolome Database and MetaboAnalyst, categorized based on published articles and KEGG analysis (Figure 8). Metabolic pathway analysis was performed using MetaboAnalyst 4.0, identifying significant pathways including Phenylalanine, tyrosine, and tryptophan biosynthesis, Phenylalanine metabolism, Aminoacyl-tRNA biosynthesis, glycerophospholipid (GPL) metabolism, glycine, serine, and threonine metabolism, and Riboflavin metabolism.

## DISCUSSION

Some studies have indicated significantly reduced total calcium concentration in hypocalcemia dairy cows compared to those that are normal at calving [16]. At the onset of lactation, Ca concentration decreases, prompting immediate secretion of PTH [17]. In our study, we found that subclinical low-calcium dairy goats not only exhibited reduced PTH level on the day of calving but also maintained lower PTH level throughout the perinatal period compared to normal dairy goats. This indicates that insufficient PTH secretion in low-calcium dairy goats impairs their ability to mobilize bone calcium and absorb intestinal calcium, resulting in delayed supplementation and hindering negative Ca feedback mechanism of PTH, which is essential for increasing blood calcium concentrations in hypocalcemic cows [18]. Thus, perinatal dairy animals needs to mobilize the secretion of PTH to restore the plasma calcium concentration due to reduced calcium ions before and after calving. CT would resist the release of PTH with the increase in Ca concentration [19]. Our results found that from one week before delivery to one week after, subclinical low-calcium dairy goats consistently showed increasing levels of 1,25(OH)_2_D_3_, PTH, and CT hormones over time. We reasonably speculated that in SCHC dairy goats, PTH concentration would increase with the decrease of Ca caused by delivery, However, its synthesis may be blocked, preventing blood calcium levels from returning to the normal. In this study, most amino acids in the SCHC group were found to be lower than those in the healthy group, suggesting increased utilization of amino acids in the low-calcium dairy goat group. In other studies, aromatic amino acid metabolism (especially tyrosine, phenylalanine, and tryptophan) may have dysfunction in SCHC [20]. Notably, Phenylalanine metabolism emerges as a crucial pathway affecting the SCHC group, with lower levels observed compared to healthy dairy goats. Previous studies have shown that in the SCHC group, after liver damage, the citric acid cycle in the liver is inhibited, and the inactivation and clearance capacity of Aromatic amino acid is reduced, resulting in a significantly higher plasma Aromatic amino acid in the SCHC group than in the healthy group [12]. The results in this study also prove that in the SCHC group, riboflavin phenylalanyl glutamic acid, phenylalanine, tyrosine, etc. in SCHC group are significantly different from the HEAL group [20]. Previous reports on cow health and subclinical studies reported that low calcium affects blood coagulation and complement pathways. It is known that proteins involved in the complement and coagulation pathway, including serine and complement factors, are highly dependent on plasma Ca concentration [21,22], which is a crucial factor for coagulation. In this study, serine, as a coagulation pathway protein, was found to be mobilized in the plasma of the SCHC group. We speculate that in SCHC dairy goats, the rapid drop in calcium ion concentration leads to disorder in the regulation of blood coagulation system causing PS, which is usually intracellular, to become more exposed in plasma for regulatory purposes. Nevertheless, due to suboptimal calcium ion levels, the exposed serine fails to activate coagulation factors. SCHC may disrupt key metabolism and immune pathways of dairy cows, leading to decreased production performance and immunity. Since serine plays a pivotal role as an amino acid in the complement coagulation system and immune regulation, these observed alterations assume significance in comprehending the initiation and advancement of SCHC and related disorders.

In this study, proteomics and lipidomics have shown that GPL metabolism bring about important changes in SCHC. As the main component of biomembranes [23], GPL significantly influence membrane stability and permeability. Recent work indicates that membrane phosphoinositol is a key factor determining the activity of the Ca^2+^ channel [24]. Inositol functions as a second messenger in neuronal signal transduction and osmotic regulation, mobilizing intracellular calcium [25]. Previous research has indicated the beneficial impact of inositol under pathological conditions like hypothyroidism, weight gain, and cardiac function recovery, with these conditions linked to inositol depletion. PI acts as the second messenger for calcium mobilization, facilitating the transmembrane transport of intracellular Ca ions. Due to the concentration of Ca ions related to smooth muscle contraction, vascular smooth muscle tension can be regulated by a series of agonists that act through receptor-mediated transmembrane signaling pathways to alter the concentration of key intracellular second messengers [26]. Therefore, for post-parturient dairy goats, supplements high in PI and high in GPL may be a better choice.

## CONCLUSION

In conclusion, non-targeted metabolomic and lipidomic analyses have unveiled significant differences between healthy dairy goats and those affected by SCHC. Our investigation into the impact of SCHC on calcium homeostasis and disease pathogenesis revealed several key insights. Specifically, PTH levels were consistently lower in SCHC goats from one week before one-week post parturition, indicating that PTH administration prior to parturition could prevent SCHC. Furthermore, abnormal phenylalanine metabolism, which may impair vitamin D synthesis and calcium absorption, suggests that monitoring phenylalanine level may provide insights into disease pathogenesis. Lipidomic analysis also demonstrated elevated PI concentrations in SCHC goats, likely due to its role in calcium ion mobilization, pointing to a heightened demand for calcium and the potential of PI as a biomarker. Collectively, these findings underscore the potential of phenylalanine, PTH, and PI as biomarkers for early detection and management of SCHC, contributing to the development of strategies to enhance dairy goat health and performance.

## Figures and Tables

**Table 1:** Comparison of plasma biochemical indexes of perinatal subclinical hypocalcemia and healthy dairy goats on the day of delivery^1)^

Biomarker	HEAL	SCHC	p-value^2)^
BHBA (mmol/L)^3)^	0.39±0.09	0.56±0.065	<0.0001
AST (U/L)^3)^	77.78±34.04	125.3±26.91	0.004
ALP (U/L)	157.1±89.87	110.2±83.43	0.268
LDH (U/L)	280.2±60.68	327.6±114.8	0.291
NEFA (mmol/L)	0.55±0.41	0.78±0.45	0.292
TC (mmol/L)	1.933±0.47	2.10±0.31	0.384
GLB (g/L)	41.56±7.47	44.22±6.96	0.445
LDL (mmol/L)	0.55±0.16	0.62±0.19	0.466
CREA (μmol/L)	46.00±9.17	48.67±10.68	0.578
HDL (mmol/L)	1.24±0.29	1.30±0.14	0.589
GLU (μmol/L)	4.16±1.93	4.91±4.07	0.622
TG (mmol/L)	0.16±0.06	0.14±0.054	0.688
UREA (μmol/L)	4.92±1.38	4.68±1.73	0.756
ALB (g/L)	27.00±5.37	26.47±4.30	0.819
ALT (U/L)	16.22±3.38	16.67±5.10	0.83
GGT (U/L)	50.67±11.05	49.78±14.09	0.883
CHE (U/L)	112.9±30.43	112.0±21.77	0.944
TP (g/L)	68.42±12.29	68.24±10.28	0.974
Ca (mmol/L)^3)^	2.36±0.11	2.09±0.07	<0.0001
1,25(OH)_2_D_3_ (ng/L)	97.37±3.918	83.41±8.19	0.1847
PTH (ng/L)	112.7±15.16	97.16±10.88	0.4146
CT (ng/L)	645.2±87.1	579±41.04	0.5069

1) HEAL goats, n = 9; SCHC goats, n = 9 (mean±standard deviation).

2) Values were calculated from unpaired t-tests. *p<0.05 by unpaired t-tests.

3) p<0.01 indicates highly significant differences between groups.

Significant differences were indicated by ** p<0.001 and *** p<0.0001.

HEAL, healthy; SCHC, subclinical hypocalcemia; BHBA, β-hydroxybutyrate; AST, glutamic oxaloacetic transaminase; ALP, alkaline phosphatase; LDH, lactate dehydrogenase; NEFA, non–esterified fatty acids; TC, total cholesterol; GLB, globulin; LDL, low-density cholesterol; CREA, creatinine; HDL, high-density cholesterol; GLU, glucose; TG, triglyceride; UREA, Synonyms; ALB, albumin; ALT, aminotransferase; GGT, γ-glutamyl transpeptidase; CHE, cholinesterase; TP, total protein; Ca, calcium; 1,25(OH)2D3, 1,25-dihydroxy vitamin D3; PTH, parathyroid hormone; CT, calcitonin.
